# Towards the use of hydrogels in the treatment of limbal stem cell deficiency

**DOI:** 10.1016/j.drudis.2012.07.012

**Published:** 2013-01

**Authors:** Bernice Wright, Shengli Mi, Che J. Connon

**Affiliations:** 1 University of Reading, School of Pharmacy, Reading, Berkshire, RG6 6UB, UK; 2 Tsinghua University, Graduate School, Shenzhen, China

## Abstract

Corneal blindness caused by limbal stem cell deficiency (LSCD) is a prevailing disorder worldwide. Clinical outcomes for LSCD therapy using amniotic membrane (AM) are unpredictable. Hydrogels can eliminate limitations of standard therapy for LSCD, because they present all the advantages of AM (i.e. biocompatibility, inertness and a biodegradable structure) but unlike AM, they are structurally uniform and can be easily manipulated to alter mechanical and physical properties. Hydrogels can be delivered with minimum trauma to the ocular surface and do not require extensive serological screening before clinical application. The hydrogel structure is also amenable to modifications which direct stem cell fate. In this focussed review we highlight hydrogels as biomaterial substrates which may replace and/or complement AM in the treatment of LSCD.

## An overview of LSCD therapy

**Figure 1:**
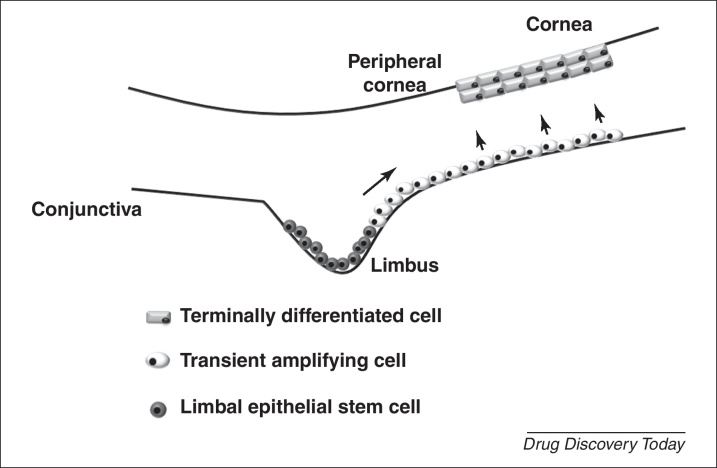
The limbal stem cell niche. Corneal stem cells reside in the limbus at the corneoscleral junction between the conjunctiva and the cornea. Epithelial stem cells in the basal region of the limbus regenerate the corneal surface by differentiating into transient amplifying epithelial cells, which give rise to terminally differentiated epithelial cells that populate the suprabasal and superficial layers of the cornea. Damage to the limbal stem cell niche results in LSCD, disrupting regeneration of the corneal epithelium.

Damage to the outer limbal region of the cornea due to chemical (e.g. acid and alkali burns) and mechanical (e.g. extended contact lens wear) injuries, congenital disorders (e.g. Stevens Johnson syndrome) or bacterial and viral infections, causes destruction and depletion of resident adult stem cells [1–5]. LSCD is manifested by conjunctival and epithelial ingrowth, vascularisation, chronic inflammation, recurrent erosions, persistent ulcers, destruction of the basement membrane (BM) and fibrous tissue ingrowth [2–5]. These pathologies lead to severe functional impairment of the cornea and clinical symptoms include irritation, epiphora, blepharospasms, photophobia, pain and decreased vision [2–5]. Consequently, disruptions in renewal of the corneal epithelium occur (Fig. 1), which ultimately leads to blindness, and this is complicated by scarring, inflammation, and the invasion of conjunctival tissue [2–5].

A diverse range of clinical methodologies, presenting inherent benefits and limitations, are currently available for treating LSCD [2–10]. Accurate diagnosis of the extent of LSCD (partial or total) is crucial for the planning of effective strategies to treat this condition. Variations in the severity of LSCD, however, indicate that the application of one single treatment type will not be sufficient for all indications of this disorder. We will therefore present a concise, critical evaluation of the evolution of LSCD therapy to appreciate the range of treatment modalities for this disorder; we refer readers to a more comprehensive review by Tseng *et al*. [5] summarising treatments for this condition.

The basis for LSCD therapy is the transplantation of progenitor limbal epithelial cells (LEC) into the damaged cornea. The foundations of modern treatment approaches for LSCD were laid when a surgical procedure using autologous conjunctival limbal autografts for contralateral cases of this condition was described [3]. This procedure restored corneal epithelial phenotype and reduced levels of goblet cells in the recipient cornea. Therapy for bilateral LSCD was subsequently performed with the transplantation of limbal allografts from cadavers, and was demonstrated as a viable strategy for reconstruction of corneal surfaces that had undergone bilateral diffuse destruction, with the loss of limbal stem cells (LSC) [4].

Therapy for LSCD progressed with the use of amniotic membrane (AM). AM alone was demonstrated as sufficient to treat partial (i.e. less than 360 degree damage to limbal tissue) LSCD [6], and AM without a limbal allograft or autograft has proven successful therapy for ocular damage as severe as chemical burns [6].

The main clinical benefits of AM include the ability to promote epithelialisation [6], reduce pain and scarring [7], and minimise inflammation [8]. The effectiveness of this biological substrate for promoting the success of transplanted LEC to treat total LSCD, led to AM becoming the scaffold of choice for *ex vivo* expansion of LEC, a technique that eliminates the need for removing the limbus from a healthy eye [9]. Methodology for the cultivation of LEC on AM to preserve their stem phenotype and encourage proliferation and stratification continues to be rigorously investigated [5,10]. The preparation of cells [as an outgrowth from biopsies (explants) or isolated into a single-cell suspension] and the use of growth factors alone or from feeder cells (e.g. 3T3 fibroblasts) are examined as conditions that promote LEC growth and progenitor phenotype [5]. LEC culture on intact (iAM) or devitalised and/or denuded (dAM) AM and exposure of cultivated LEC to the air–liquid interface (air-lifting) are also studied to develop methods that maintain LEC stemness and stratification, respectively [5]. Furthermore, studies are underway to modify the structure of AM to enhance the ability of this substrate to support the expansion of LEC [10].

Key problems encountered with LSCD therapy that limit therapeutic outcomes include: (i) variations in treatment outcomes due to structural heterogeneity of AM scaffolds and differences in the severity of LSCD, (ii) a poor understanding of mechanisms underlying corneal repair mediated during LSCD therapy, (iii) limited supplies of donor tissue which is essential in cases of bilateral total LSCD and (iv) inadequate means for eliminating long-term immunosuppression that is necessary following transplantation of allogeneic sources of LEC.

Solutions proposed for these challenges include: (i) the use of alternative cell types, corneal prostheses and cell storage strategies to alleviate problems with limbal tissue availability, (ii) the use of structurally uniform biomaterials for the delivery of LEC to the damaged ocular surface to eliminate variations in LSCD treatment and (iii) the use of biomaterials with well-characterised and easily modified structures to understand mechanisms by which LEC reverse the symptoms of LSCD.

## Biomaterials for regeneration of the cornea

The main uses for biomaterials applied to reconstruction of damaged corneal epithelium include hydrogels for LEC delivery [11–19], bioengineered prosthetic devices (keratoprostheses: KPro) that replace dysfunctional corneal tissue [20–26], contact lenses used as ocular bandages or for the correction of refractive errors [27–29], and materials for transcorneal drug delivery [30–34]. Ocular biomaterials used for the fabrication of intraocular lenses, glaucoma filtration implants, scleral buckles and viscoelastic replacement agents are reviewed elsewhere [35] and will not be discussed in this article. The wide range of biomaterials currently in use for regeneration of the cornea includes hydrogels [11–19,36,37], porous silk fibroin films [38], keratin from hair or wool [39], 3D nanofibre scaffolds fabricated from polyamide 6/12 (PA6/12) [40] and electrospun poly(lactide-co-glycolide) membranes [41].

Structural modification of biomaterials to enhance LEC adhesion [42,43] and control the differentiation [44] of these cells is the current direction for the application of these tools. Corneal epithelial tissue grown on polycarbonate surfaces with pore diameters of 0.1–3.0 μm were shown to lay down continuous BM and a regular pattern of hemidesmosomal plaque on a 0.1 μm surface, and no adhesive structures assembled on a nonporous or 3.0 μm surface [42]. On hydroxyethylmethacrylate (HEMA) hydrogels modified by the addition of amines (N,N-dimethylaminoethylmethacrylate) or carboxyl moieties (methacrylic acid), the expression of adhesion receptors, integrin α6 and β4 in corneal epithelial cells was shown to be higher on surfaces containing amine moieties than on surfaces containing only carboxyl moieties [43]. Furthermore, modification of the stiffness of collagen gels was recently shown to direct LEC differentiation [10,44].

The integration of a range of compatible biomaterials with complementary functions for the construction of artificial systems may lead to the development of sophisticated medical devices with the potential to considerably enhance the efficacy and predictability of LSCD therapy. Those systems may be designed specifically to treat the differing extents of LSCD, with combined delivery of drug and cell therapies. Hydrogels, in particular, present key properties which indicate their suitability for the culture and/or delivery of LEC (i.e. chemical inertness, uniformity of structure, biocompatibility, and mechanical strength and pliability).

**Figure 2:**
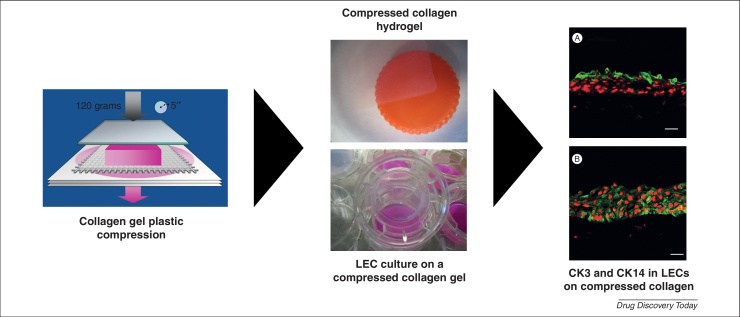
The use of a compressed collagen hydrogel for *ex vivo* expansion of limbal epithelial cells. Collagen is plastically-compressed using a 120 g load. CK3 **(A)** and CK14 **(B)** are expressed in LEC (green) expanded on a laminin-coated compressed collagen gel embedded with keratocytes. Cell nuclei are stained with propidium iodide (red). Images represent 3 different experiments from 3 different corneoscleral rims. Scale bar: 50 μm. Reproduced from ref. [24].

Hydrogels for the design of corneal prosthetics are also becoming increasingly popular. Collagen [20–26] and poly(2-hydroxyethyl methacrylate) (PHEMA) [45] gels are mainly applied to the construction of KPro that are biointegrable in a manner that promotes regeneration of corneal cells, nerves, and extracellular matrix (ECM). In our own laboratory we have characterised laminin-coated, plastically compressed collagen gels containing corneal fibroblasts suitable for the delivery of LEC to the damaged cornea (Fig. 2) [24–26].

In this article we highlight several well-established and emerging hydrogel systems and discuss their suitability for treating LSCD.

## The potential for the use of hydrogels in LSCD therapy

Hydrogels are multi-component systems consisting of a 3D network of polymer chains and water [46]. Physical gels (pseudogels) comprise chains of macromolecules that are connected by electrostatic forces, hydrogen bonds, hydrophobic interactions or chain entanglements, and in chemical hydrogels, covalent bonds link polymer chains. Hydrogels are attractive scaffolding materials owing to their highly hydrated network structure, which enables encapsulation of cells and bioactive molecules, and efficient mass transfer of soluble factors to and from immobilised cells [46]. Clinical and pre-clinical studies indicate that fibrin, collagen, silicone, alginate, chitosan and gelatin hydrogels are lead candidates for the treatment of LSCD (Table 1).

### Fibrin hydrogels

Fibrin gels are prepared by the combination of fibrinogen and thrombin, or from autologous serum [47]. These hydrogels are used extensively as biopolymer scaffolds to regenerate adipose tissue, bone, cartilage, cardiac tissue, liver, nervous tissue, ocular tissue, skin, tendons and ligaments [47]. Certainly, the use of fibrin hydrogels in LSCD therapy is already established [11–15].

Previous reports demonstrated that the majority of individuals within a group presenting LSCD regained their vision following treatment with fibrin-cultured LEC, and this outcome was sustained over long-term periods [12,13]. Rama *et al*. [1,12] reported a study where 14 out of 18 patients treated with LEC cultured on fibrin gels rapidly regained useful visual acuity. The corneas of treated patients underwent re-epithelialisation within the first week, inflammation and vascularisation was reduced within the first three to four weeks, and at a 12–27 month follow-up, corneal surfaces were clinically and cytologically stable [12].

Subsequent studies demonstrated that fibrin gels were capable of supporting the stem and/or progenitor phenotype of LEC [13,14]. These gel scaffolds preferentially cultivated the expansion (*ex vivo*) of LEC; subcultivation of limbal holoclones preserved stem and progenitor cells in the basal layer of fibrin-based epithelial sheets [14]. Furthermore, the generation of normal, renewing epithelium on donor stroma treated with fibrin-cultured LEC correlated with cultures in which p63 bright cells constituted more than 3% of the total number of clonogenic cells [13]. The majority (78%) of LSCD patients treated with fibrin-cultured LSC containing ≥3% p63 bright cells were successfully treated, whereas only 11% of LSCD patients received viable treatment with cultures containing ≤3% p63 bright cells [13].

Interestingly, another progenitor cell type, mesenchymal stem cells (MSCs), encapsulated in fibrin gels induced reconstruction of the damaged corneal surface, and these cells expressed the corneal epithelial cell specific marker, cytokeratin 3 (CK3) when they were transplanted [15]. Further investigation under *in vitro* conditions demonstrated that MSCs co-cultured with LEC or LEC conditioned medium, rapidly differentiated into cells that were phenotypically and morphologically similar to corneal epithelial cells [15]. These results are supported by a recent report demonstrating that MSCs reside in the limbal niche [48].

Therefore, fibrin hydrogels are clearly capable of maintaining the phenotype and directing the fate of stem cells, indicating that they may be exploited to understand the fundamental biology of LSC differentiation and self-renewal. The positioning of limbal holoclones on fibrin-cultured epithelial cells [14] suggests that these biomaterials may be used to construct a niche-like environment for LSC. Certainly, there is considerable potential to begin understanding mechanisms that regulate LSC function during transplantation, using these gels as *in vitro* systems. The requirement for effective logistics to deliver LSCD therapy together with the practicality of fibrin-cultured LEC, suggest that this hydrogel can form part of cell preservation and transportation technologies for wide ranging applications in corneal reconstruction therapies.

### Collagen hydrogels

Collagen is the most abundant structural protein in the cornea, and is biocompatible, biodegradable, possesses low immunogenicity and can maintain LEC adhesion, proliferation and differentiation [20–26]. The BM of the limbal epithelium contains type IV collagen (α1 and α2 chains) as well as type IV collagen (α3 chain), and type V collagen is present in the corneal BM [49]. At present, collagen hydrogels are mainly applied to LEC culture [16] and the formation of tissue-engineered scaffolds or KPro used for replacement of corneal tissue [20–26].

Magnetically oriented collagen fibre scaffolds were recently applied to the regeneration of human hemi corneas *in vitro* [23]. Reconstruction of the hemi cornea involved the formation of a well-defined epithelium as well as stroma by keratocytes which when aligned with collagen, were shown to lay down ECM components with features typical of collagen fibrils [23]. The aligned collagen and/or keratocyte construct induced re-epithelialisation of corneal stroma *in vivo* in a rabbit corneal injury model [23], indicating the potential of this biologically functional medical device for treating LSCD.

By contrast, conventional collagen hydrogels are plastically compressed or chemically cross-linked to enhance mechanical strength before their use in the formation of stratified LEC [16,24–26]. This strategy is in development at present, to design corneal equivalents for application as biointegrable KPro.

1-Ethyl-3-(3-dimethyl aminopropyl) carbodiimide (EDC) and N-hydroxysuccinimide (NHS) cross-linked recombinant human collagen-based artificial corneas are one of the first examples of these constructs tested on humans. In a Phase I clinical trial, this biosynthetic scaffold was transplanted to the damaged cornea of ten patients with vision loss [22]. Six-month postoperative results demonstrated the regeneration of host epithelium and the growth of stromal cells into the implant, and a 24-month follow-up report of this study showed that implants retained stability and remained avascular without prolonged use of steroid immunosuppression, commonly required in traditional allotransplantation. Corneal re-epithelialisation, tear film formation, stromal cell maturation and nerve regeneration was observed in all patients and after 24 months, vision was significantly improved in six patients [22]. This study was promising because it indicated that collagen KPro can be applied as donor corneas in a clinical setting.

Simultaneous use of collagen hydrogels as corneal tissue replacements and as delivery systems for LEC may lead to the development of KPro capable of treating LSCD.

### Silicone hydrogels

Silicone hydrogels comprise lotrafilcon A, balafilcon A, senofilcon A and comfilcon A, and are mainly used to produce second generation, soft contact lenses for the correction of refractive errors and as ocular bandages which aid re-epithelialisation of the cornea [27]. The major advantage of silicone lenses is efficient transmission of oxygen to the ocular surface using its water and polymer content [27]. Highly oxygen-permeable (Dk) silicone hydrogel lenses enable rapid and stable re-epithelialisation of the cornea, have eliminated lens-induced hypoxia for the majority of wearers and have a less pronounced detrimental effect on corneal homeostasis compared to other lens types [28].

Recent reports suggest that in addition to application as a visual aid and an ocular bandage, silicone hydrogels may be applied to LSCD therapy [29,30]. The treatment of persistent corneal defects (PED), which include LSCD, dry eye syndrome, graft-versus-host disease, toxic keratitis, limbic keratoconjunctivitis, and neurotrophic keratitis [29], with hydrogel contact lenses (i.e. silicone lenses) is well-documented [27–30]. Moreover, lotrafilcon A contact lenses were reported to sustain proliferation and migration of LEC from limbal tissue, which displayed a corneal phenotype (CK3^+^/CK12^+^/CK19^+^) and expressed p63 [30]. Microvilli with adhesive projections were observed on the apical surface of LEC cultured on the lens indicating that these cells were stable and likely to survive long-term [30]. Therefore, these findings suggested that it may be possible to generate a corneal epithelium on silicone contact lenses and easily transfer this to cornea presenting LSCD.

Despite the therapeutic benefits of silicone lenses for PED and LSCD, further research is needed to improve the biocompatibility of this biomaterial. The high modulus (silicone hydrogels are stiffer and less flexible than conventional hydrogels) of these lenses causes mechanical interaction with ocular tissue which lead to papillary conjunctivitis and disruption of the tear film structure [27]. Furthermore, the high oxygen permeability of silicone lenses induces epithelial inclusions related to mucin ball formation [50]. The development of silicone contact lens systems for the expansion and delivery of LEC will therefore require investigations into structural modifications (i.e. alterations to modulus and Dk) of this hydrogel which favourably complement corneal homeostasis and physiology.

### Alginate hydrogels

Alginates are polysaccharides found in brown algae and bacteria, consisting of unbranched binary co-polymers of 1–4 linked β-d-mannuronic acid and α-l-guluronic acid, which when cross-linked by multi-valent cations (e.g. Ca^2+^, Ba^2+^, La^3+^, Fe^3+^, Zn^2+^, Mg^2+^, Sr^2+^) form into gels [50]. Alginate hydrogels are one of the most well-characterised cell immobilisation substrates [51,52] due to effective immunoisolatory and mass transfer properties [50], and are widely used as biomaterial tools in the field of regenerative medicine [53–55].

The use of alginate hydrogels for ocular therapy is relatively novel. Only a small number of studies have been performed to examine the manner that alginate may be applied for ocular reconstruction. Alginate microspheres incorporated into collagen hydrogels were previously demonstrated as a viable composite construct for controlled drug delivery as well as human corneal epithelial cell growth [31]. Alginate membranes coated with chitosan used as base matrices for LEC cultivation maintained the attachment, spreading and growth of these cells [18]. Another composite hydrogel containing sodium alginate dialdehyde and hydroxypropyl chitosan was used for transplantation of corneal endothelial cells (CEC) onto Descemet's membrane, and demonstrated that encapsulated CEC remained viable and retained their normal morphology [56].

**Figure 3:**
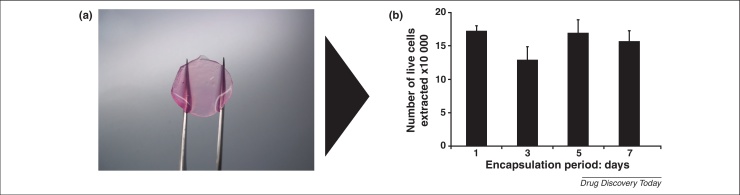
Alginate gels as LEC storage devices. Calcium alginate gel discs **(a)** with dimensions approximately 19 mm in length and 1.5 mm in depth are viable storage modules for LEC. Images (100× magnification) represent three individual experiments. Data points on bar chart **(b)** represent the mean (*n* = 3 ± S.E.M.) number of live cells extracted from alginate gel discs following 1, 3, 5 and 7 day culture periods.

These preliminary studies have highlighted the potential of alginate gels as culture systems for LEC. Because the structure of alginate gels can be modified to direct cell phenotype, particularly stem cell differentiation [57,58], and enhance cell survival, this biomaterial presents an ideal culture system for LEC, which may be developed for application to reversing the pathology of LSCD. Moreover, the well-reported use of alginate gels for cell preservation [59,60] will allow this hydrogel to be developed into a transport and/or storage medical device (Fig. 3) applicable to corneal cell-based therapies.

### Chitosan hydrogels

The mucoadhesive polysaccharide, chitosan is biocompatible, biodegradable and displays unique haemostatic activity and wound-healing properties that makes it attractive for use in biomedical applications [19]. Chitosan alone was previously applied topically to the eyes of rabbits to repair the endothelium after they had sustained central corneal wounds, because these polymers were indicated to induce regeneration of vascular endothelium [36].

Synthetic chitosan membranes supported the viability and growth of corneal epithelial cells in a manner comparable to AM [19]. This study was supported by another report showing that chitosan membranes modified with poly-d,l-lactic acid promoted wound healing of alkali-burned corneas *in vivo* and decreased scar tissue formation [37]. These findings are particularly exciting because one of the major advantages of AM is its anti-scarring abilities. Therefore, chitosan may be a substitute for AM.

The well-characterised abilities of chitosan for ophthalmic drug delivery may be exploited together with the capacity of this polymer for cultivation of corneal epithelial cells. Chitosan polymers can increase precorneal drug residence times to slow down drug elimination by the lacrymal flow, by increasing solution viscosity and by interacting with the negative charges of the mucus [30]. Chitosan nanoparticles are also able to enhance the therapeutic index of clinically challenging drugs [32]. Chitosan hydrochloride increased transcorneal penetration of topically applied ofloxacin and the therapeutic efficacy of this ophthalmic drug [33]. Furthermore, a novel copolymer, poly(N-isopropylacrylamide)-chitosan, was previously suggested as a potential thermosensitive *in situ* gel-forming material for ocular drug delivery [34].

Chitosan hydrogels may therefore be used to create a device which delivers both LEC and ocular drugs that enhance the effects of these cells, thus constituting a novel strategy to treat LSCD.

### Gelatin hydrogels

Gelatin is a mixture of peptides and proteins produced by acid and alkaline processing (partial hydrolysis) of collagen extracted from skin, bone and connective tissue [61]. Currently, gelatin hydrogels are under development as substrates for CEC and stromal cell culture, but they have not been used as supports for cultivating LEC. These gels have received the most attention for ocular use as drug delivery vehicles.

The success of gelatin gels for the culture of CEC and stromal cells indicate that LEC may be well-supported by these scaffolds. Gelatin gels were previously shown to provide stable mechanical support for CEC sheets [62], enabling expression of typical markers for these cells [zonula occludens-1, Na^+^/K^+^-ATPase, and N-cadherin] [63]. Porous gelatin was demonstrated as suitable for the engineering of corneal stroma [64].

Soluble ocular drug insert matrices comprised of raw gelatin [65–68] or chemically modified [69,70] and composite gelatin [71,72] gels also indicate the feasibility of these biomaterials for LEC culture. Gelatin gels cross-linked with EDC were shown to support the culture of iris pigment epithelial cells more efficiently than gels cross-linked with glutaraldehyde [69]. A cationised gelatin film with incorporated epidermal growth factor (EGF) resulted in a reduction in an epithelial defect in rabbit corneas; this was accompanied by significantly enhanced epithelial proliferation compared with the reduction observed with topical application of EGF solution or the placement of an EGF-free gelatin film [65]. Polyvinyl alcohol (PVA)–gelatin polymeric blends are also promising as ocular inserts for prolonged release of antibiotics in the eye [71].

The absorbable gelatin sponge, Gelfoam^®^, is a manufactured drug carrier for either local or systemic drug delivery through the ophthalmic route. This eye medical device was reported to prolong the activity of the insulin through gradual release aided by the slow and constant tear production from the lachrymal system [73]. Another study showed that dilation of the pupil induced by phenylephrine and tropicamide delivered using Gelfoam^®^ was greater and longer lasting than that produced by eyedrops with an equivalent amount of these drugs [74]. As Gelfoam^®^ can be worn with contact lenses, in a hydrated form; this hydrogel as well as other gelatin hydrogels may serve as a growth factor and/or drug-release feeder device to maintain LEC delivered to the ocular surface using silicone lenses.

### Emerging hydrogel systems

Hydrogels at the early experimental stage before application as substitutes for AM include hyaluronic acid (HA) [75] gels, thermosensitive gels (e.g. Mebiol^®^) [76,77], poly(2-hydroxyethyl methacrylate (PHEMA) [78–80] and PVA [81,82] gels that are established as component parts of the artificial cornea, as well as muscle-derived myogel [83].

HA gels chemically cross-linked with poly(N-2-hydroxyethyl) (2-aminoethylcarbamate)-d,l-aspartamide (PHEA-EDA) were previously suggested as a suitable material for the release of limbal cells for corneal regeneration [75]. HA and/or PHEA-EDA films enabled moderate and/or poor adhesion of human corneal epithelial cells, rabbit limbal epithelial cells and fibroblasts. Contact lenses coated in their inner surface with the HA/PHEA-EDA film enabled greater cell adhesion, but this was transitory; viable cells were released after three days [75]. Therefore, HA and/or PHEA-EDA hydrogels were suggested as suitable for delivering limbal cells to treat corneal damage.

The thermosensitive, synthetic polymer gel, Mebiol^®^ was previously reported as capable of supporting LEC and maintaining the stem phenotype of these cells. Mebiol^®^ supported limbal explant proliferation and LEC cultured on this gel expressed LSC markers (ATP-binding cassette sub-family G member 2 and p63), transient amplifying cell markers (connexin 43, integrin α9) and cornea differentiation marker (CK3) [76]. The transplantation of autologous LEC grown in Mebiol^®^ was shown to restore a nearly normal ocular epithelial surface in eyes with unilateral LSCD in rabbit models [77], indicating that this hydrogel is almost at the pre-clinical stage.

By contrast, PHEMA sponges used to construct the porous skirt material in the Chirila KPro require improvements in biocompatibility [78–80], to prevent calcification and proangiogenic effects before they can be developed into a viable substrate for LEC. Another biomaterial component of the artificial cornea, the porous nano-hydroxyapatite and/or poly (vinyl alcohol) hydrogel [81], may also be suitable for LEC culture; this material was demonstrated to support the maintenance and growth of corneal epithelium *in vitro* [82].

The novel muscle-derived hydrogel, myogel [83] may also provide an alternative cell (*ex vivo* expanded cells) carrier for LSCD with a further reduction in risk, as it is derived from an autologous muscle biopsy.

## Concluding remarks

We have already crossed the threshold for major change in LSCD therapy from conventional AM-based methodologies to more versatile and practical methods involving natural and synthetic biomaterial systems.

Fibrin and collagen hydrogels have developed beyond the pre-clinical stage, and they are currently proven as viable for the treatment of LSCD and as corneal prosthetics, respectively. Other hydrogels present unique properties including intrinsic anti-scarring capabilities (chitosan), efficient mass transfer abilities (alginate, gelatin, silicone) and properties appropriate for tissue engineering (alginate), which indicate them as excellent candidates for the treatment of LSCD.

The structures of ‘clean’ hydrogel systems may be manipulated to alter their physical and chemical properties, internal porosity, and surface topography to induce predictable changes in cell behaviour. Therefore, these biomaterials applied to the treatment of LSCD may potentially enable a more in-depth understanding of the mechanisms underlying the reversal of pathological symptoms of this disorder, which may introduce a novel field of ophthalmic medicine.

We conclude that given the considerable body of evidence demonstrating the efficacy of hydrogels in reconstruction of the damaged cornea, AM can certainly be replaced or complemented with these biomaterials for the treatment of LSCD.

## Conflicts of interest

None.

## Figures and Tables

**Table 1:** A summary of hydrogels at the clinical and pre-clinical stages of therapy for corneal regeneration

Hydrogel	Description	Ocular use	Clinical success	Refs
Fibrin	Fibrin hydrogels are composed of a cross-linked fibrin network either formed by the combination of fibrinogen and thrombin, or isolated from autologous serum.	*Ex vivo* expansion of LESC and encapsulation of MSC for treating LSCD.	LSCD symptoms were reversed in human patients and animal models of LSCD.	[11–15]
Plastically compressed collagen	Collagen is an ECM protein. Conventional collagen gels are inherently weak due to high water content. Therefore, they are plastically compressed to achieve a stronger gel by expelling water.	LEC culture for application to construction of an artificial cornea.	N/A.	[16,24–26]
EDC and NHS cross-linked recombinant human collagen	N/A.	Corneal epithelial cell culture.	Tested on humans in a Phase 1 clinical trial.	[22]
Recombinant human collagen-phosphorylcholine (RHCIII-MPC) hydrogels	Biosynthetic implants were fabricated from freeze-dried recombinant human collagen type III (RHCIII), either with or without the incorporation of 2-methacryloyloxyethyl phosphorylcholine (MPC).	Corneal substitute.	Promoted cell and nerve repopulation and enhanced resistance to neovascularisation in alkali-burned rabbit eyes.	[20]
Hydrated collagen and *N*-isopropylacrylamide copolymer-based ECMs	Gels were grafted with the laminin adhesion pentapeptide motif, YIGSR.	Keratoprosthesis or artificial cornea.	Successful *in vivo* regeneration of host corneal epithelium, stroma, and functional nerves in pig models.	[21]
Silicone	Silicone hydrogels are polymers composed of carbon, hydrogen and oxygen.	Soft contact lens, ocular bandage for treating persistent epithelial defects (PED) and substrate for LEC culture.	Silicone contact lens can deliver LEC to the cornea and relieve PED symptoms in humans.	[29,30]
Alginate	Alginate is a polysaccharide. Alginate hydrogels comprise blocks of mannuronic and guluronic acid cross-linked via carboxyl groups with multi-valent cations.	LEC storage.	N/A.	[59]
Alginate microspheres incorporated into collagen hydrogels	N/A.	Drug delivery and LEC culture.	N/A.	[31]
Chitosan	Chitosan is a polysaccharide. Hydrogels are produced through cross-linking chitosan using glutaraldehyde, rutin or light.	LEC culture and ocular drug delivery.	Decreased ocular drug elimination time.	[19,30,32]
Chitosan membranes modified with poly-d,l-lactic acid (PDLLA)	N/A.	Ocular bandage.	Promoted wound healing in animal models of LSCD.	[37]
Chitosan hydrochloride (CH/HCl)	N/A.	Ocular drug delivery.	Increased transcorneal penetration of ocular drugs.	[33]
(PDLLA/chitosan) membranes	Poly-d,l-lactic acid (PDLLA) was modified with chitosan.	Corneal wound healing.	Promoted wound healing of alkali-burned corneas *in vivo* and decreased scar tissue formation in rabbit models.	[34]
Gelatin	Partially hydrolysed collagen.	Corneal endothelial and stromal cell culture, and ocular drug delivery.	Supports stromal regeneration in animal model.	[62–67]
Gelfoam^®^	Gelatin sponge.	Ocular drug delivery.	Increases drug-release time.	[73,74]
Poly(2-hydroxyethyl methacrylate) (PHEMA) hydrogels	PHEMA gels are produced by mixing 2-hydroxyethyl methacrylate (HEMA) in the presence of water (HEMA/water ratio 20/80 w/w), 0.1 wt% (of monomer) cross-linking agent, and 0.12 wt% (of monomer) initiators (ammonium persulphate and tetramethylethylenediamine).	Keratoprosthesis or artificial cornea.	Supports corneal wound healing in animal models.	[45]
